# AI4AMP: an Antimicrobial Peptide Predictor Using Physicochemical Property-Based Encoding Method and Deep Learning

**DOI:** 10.1128/mSystems.00299-21

**Published:** 2021-11-16

**Authors:** Tzu-Tang Lin, Li-Yen Yang, I-Hsuan Lu, Wen-Chih Cheng, Zhe-Ren Hsu, Shu-Hwa Chen, Chung-Yen Lin

**Affiliations:** a Institute of Information Science, Academia Sinica, Taipei, Taiwan; b TMU Research Center of Cancer Translational Medicine, Taipei Medical University, Taipei, Taiwan; Dalhousie University

**Keywords:** antimicrobial peptide, protein-encoding method, deep learning, real-world data, web service

## Abstract

Antimicrobial peptides (AMPs) are innate immune components that have recently stimulated considerable interest among drug developers due to their potential as antibiotic substitutes. AMPs are notable for their fundamental properties of microbial membrane structural interference and the biomedical applications of killing or suppressing microbes. New AMP candidates must be developed to oppose antibiotic resistance. However, the discovery of novel AMPs through wet-lab screening approaches is inefficient and expensive. The prediction model investigated in this study may help accelerate this process. We collected both the up-to-date AMP data set and unbiased negatives based on which the protein-encoding methods and deep learning model for AMPs were investigated. The external testing results indicated that our trained model achieved 90% precision, outperforming current methods. We implemented our model on a user-friendly web server, AI4AMP, to accurately predict the antimicrobial potential of a given protein sequence and perform proteome screening.

**IMPORTANCE** Antimicrobial peptides (AMPs) are innate immune components that have aroused a great deal of interest among drug developers recently, as they may become a substitute for antibiotics. New candidates need to fight antibiotic resistance, while discovering novel AMPs through wet-lab screening approaches is inefficient and expensive. To accelerate the discovery of new AMPs, we both collected the up-to-date antimicrobial peptide data set and integrated the protein-encoding methods with a deep learning model. The trained model outperforms the current methods and is implemented into a user-friendly web server, AI4AMP, to accurately predict the antimicrobial properties of a given protein sequence and perform proteome screening.

**Author Video:** An author video summary of this article is available.

## INTRODUCTION

Antimicrobial peptides (AMPs) are a diverse group of small bioactive proteins originally derived from a broad spectrum of living species, including prokaryotes, plants, invertebrates, and vertebrates (1). Most discovered AMPs are cationic amphipathic small peptides and usually <100 amino acid residues in length. They act as the first-line defense molecules that kill or suppress surrounding microbes or are inferred as weapons for resource competition among microbes (2). The most widely known mechanism through which AMPs kill microbes is osmotic shock, which occurs through either the formation of pores or paving as carpet on the membrane surface to weaken membrane integrity (1). Compared with other widely used antibiotics such as penicillins, tetracyclines, and nonribosomal peptide polymyxins that exert effects on specific biological pathways, AMPs target the common physicochemical properties of biological membranes to kill microbes, thus reducing the likelihood of rapidly evolving microbial resistance. In the face of the public health crisis associated with the emergence of antibiotic-resistant bacteria (3, 4), AMPs are promising alternative candidates for infection treatment (5).

Rather than purifying AMPs from natural sources, synthesizing these small bioactive peptides at a reasonable cost is possible. Complex approaches have been adopted to increase the potency of these small peptides as drug candidates or design a new sequence *de novo* with antimicrobial activity based on previous knowledge and molecular simulation findings (6). Machine learning, especially deep learning, can extract useful information from complex data sets (7). Various learning methods have been introduced to solve AMP prediction problems. For example, the AmPEP prediction model (8) was implemented using a random forest trained in a set of AMP sequences encoded by the distribution patterns of amino acids. The iAMPpred model (9) was based on a support vector machine (SVM), and the encoding considered the residue composition and physicochemical properties as well as structural properties. Veltri et al. (10) developed the antimicrobial peptide scanner, version 2, by encoding AMPs through a neural network embedding layer and then utilized deep learning methods to develop the AMP classifier. The presence of numerous sequence candidates from predictors complicates the screening of useful AMPs. A highly accurate machine learning strategy for evaluating antimicrobial activity can balance the cost and effort.

This study used an up-to-date AMP data set with unbiased negatives and stratified it to investigate protein-encoding methods. We proposed physicochemical component 6 (PC6), a new protein-encoding method that provides six physicochemical properties of each amino acid in a peptide sequence. We found that the AMP prediction of the PC6 deep learning design performed moderately when internal testing data were used but outperformed other latest methods when external testing data were used. Furthermore, we developed a website service, AI4AMP, based on the PC6 deep learning model to accurately predict the antimicrobial properties of a given protein sequence and perform proteome screening.

## RESULTS

### Six physicochemical properties used in PC6.

**FIG 1:**
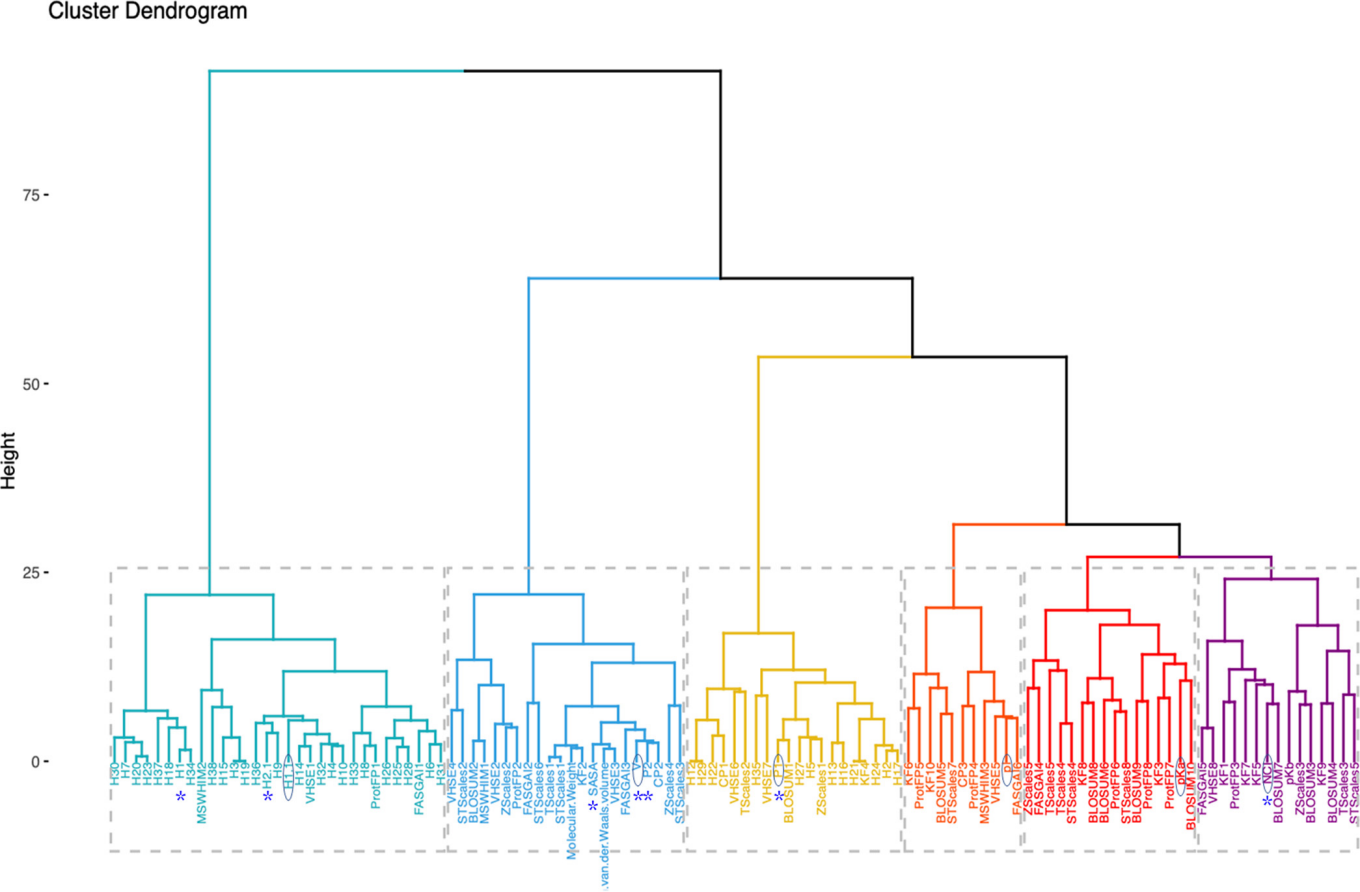
Hierarchical clustering plot of physicochemical properties. Six selected physicochemical properties used in our PC6 are marked in blue. Seven physicochemical properties used in the original AC7 method are marked with asterisks.

We recorded the physicochemical properties of amino acids by using the R package Peptides (11). The 115 properties, excluding those given a value of “NA,” were accessed to examine the relatedness through hierarchical clustering. On the basis of the within sum of squares, we selected six as the optimal cluster quantity and one property from each cluster. Based on the seven features in the original autocovariance (AC) method (12), we chose hydrophobicity (H1), the volume of side chains (V), polarity (Pl), and pH at the isoelectric point (pI) from the four clusters. As for the remaining two clusters, we selected two common physicochemical properties: the dissociation constant for the -COOH group (pK_a_), and the net charge index of the side chain (NCI) reflecting AMP’s cationic characteristic to establish our PC6 encoding method (Fig. 1). The seven features in the original AC method (13) were also included. These features were located in four of the six subclusters, especially focused in subcluster II. Notably, the features derived from sequence similarity scored on different blosum matrixes were scattered in the six subclusters.

### Performance of protein-encoding methods.

AMP and non-AMP sequences were collected and stratified into the training data set (positive, negative, and internal test sets) and the external testing data set described in Materials and Methods. We first established our PC6 encoding method (described in Materials and Methods) and tested the prepared data set’s performance. Three other peptide sequence encoding methods, namely, the AC method (13), embedding layer (14), and Word2Vec (15), were included in this study. To render the results of PC and AC methods comparable, the encoding scheme was duplicated to adopt either the six properties in the selected PC6 method or the seven physicochemical properties proposed in the original AC method (designated AC6, AC7, PC6, and PC7 accordingly). Within this framework of shared deep learning model architecture and data sets for training and testing, the performance of these encoding methods is detailed in Table 1.

**TABLE 1:** Performance of protein-encoding methods with deep learning^*a*^

Encoding method	Accuracy	Precision	Sensitivity	Specificity	F1 score	MCC
	Test data (706 sequences)					
PC6	0.8895	0.9205	0.8527	0.9263	0.8853	0.7812
PC7	*0.8782*	*0.9083*	*0.8414*	0.9150	*0.8735*	*0.7584*
AC6	*0.7040*	*0.7130*	*0.6827*	*0.7252*	*0.6975*	*0.4083*
AC7	*0.7507*	*0.7580*	*0.7365*	*0.7649*	*0.7471*	*0.5016*
Embedding layer	0.8952	0.9091	**0.8782**	*0.9122*	0.8934	0.7908
Word2Vec	**0.9065**	**0.9415**	0.8669	**0.9462**	**0.9027**	**0.8156**
	External testing data (1,130 sequences)					
PC6	**0.8850**	0.9035	**0.8620**	0.9080	**0.8822**	**0.7707**
PC7	0.8610	0.8849	0.8301	0.8920	0.8566	0.7235
AC6	*0.7818*	*0.8534*	*0.7009*	*0.8695*	*0.7697*	*0.5760*
AC7	*0.7311*	*0.8152*	*0.6248*	*0.8464*	*0.7074*	*0.4808*
Embedding layer	0.8690	**0.9096**	*0.8195*	**0.9186**	0.8622	0.7417
Word2Vec	*0.8460*	*0.8574*	0.8301	*0.8619*	*0.8435*	*0.6924*

a Top three ranked methods for each index are presented using text format: first in boldface, second with underline, third in normal text format, and all the rest in italic.

The purpose of stratifying our peptide usage was to minimize external testing data that would challenge the ability of AMP predictors. The LAMP database was stored separately from the data set used for the model construction. Hence, we used these labeled data to simulate the real-world problem because novel peptide sequences with AMP function may have never been reported. An external testing data set could indicate whether our model was effective in determining novel sequences. Furthermore, data contamination is a common problem in developing and validating machine learning models, causing the overestimation of a model’s efficacy. We excluded data if they matched each model’s training data set. Thus, this external data set was unbiased and equitable for comparing the performance of AMP predictors.

We used six measures to examine the performance of these protein-encoding models. Word2Vec outperformed other methods with the validation input set. However, Word2Vec could not maintain this predominance in the external testing data set, being outperformed by PC6. The robustness of encoding methods was determined using 10-fold cross-validation. The average accuracy was 0.87 ± 0.02, 0.88 ± 0.02, and 0.84 ± 0.06 for PC6, Word2Vec, and embedding layer, respectively.

In a biomolecule discovery study focusing on AMP prediction similar to our study, markedly fewer input data are provided compared with those used in natural language processing (NLP) studies. Word2Vec, which semantically abstracts from a literal context, performed remarkably in the model training stage but failed in the external data set. This may be due to overfitting, which is not uncommon in machine learning approaches. Embedding layers seem to fetch some other properties that Word2Vec did not achieve. Thus, the performance ranked in second place with both the internal and external data sets.

We believe that an ideal encoding method for protein sequences should consider protein features in the model, such as the physicochemical properties of amino acids derived from the independent measurement or estimation. Our PC6 encoding method performed satisfactorily with the test data set (ranked second and third for two and four performance measures, respectively [Table 1]). It was the optimal model with the external testing data set. In the cases of AC methods encoded in seven (AC7) or six (AC6) parameters as proposed in PC6, neither configuration obtained a favorable result. Although AC7 demonstrated noteworthy performance in predicting protein-protein interactions (12), it did not efficiently fit the AMP prediction task. In terms of accuracy and Matthews correlation coefficient (MCC), all encoding methods except AC7 and AC6 performed satisfactorily. One possible reason for the AC methods’ poor performance is the loss of information during the embedding process; for AMPs that are short in length (20 to 50 amino acids on average), the AC hyperparameter “lag value” is set to 10, leading to a critical loss of useful features. Notably, AC7 outperformed AC6 with the model test data set, whereas AC6 was superior with the external data set.

### Testing of traditional machine learning methods.

We applied the encoding methods with optimal performance, PC6, and Word2Vec, in two other machine learning approaches, SVM and random forest, to build models using the same data as the evaluations mentioned above. The performance of each combination is displayed in Table 2.

**TABLE 2:** Comparison of models built by different protein-encoding methods and machine learning algorithms^*a*^

Encoding/	ML algorithm	Accuracy	Precision	Sensitivity	Specificity	F1 score	MCC
		Test data (706 sequences)					
PC6/	SVM	*0.8329*	*0.8550*	*0.8017*	*0.8640*	*0.8275*	*0.6670*
PC6/	Random forest	*0.8385*	*0.8770*	*0.7875*	0.8895	*0.8299*	*0.6806*
PC6/	Deep learning	0.8895	0.9205	0.8527	0.9263	0.8853	0.7812
Word2Vec/	SVM	*0.8300*	*0.8499*	*0.8017*	*0.8584*	*0.8251*	*0.6611*
Word2Vec/	Random forest	0.8499	0.8800	0.8102	0.8895	0.8437	0.7019
**Word2Vec/**	**Deep learning**	**0.9065**	**0.9415**	**0.8669**	**0.9462**	**0.9027**	**0.8156**
		External testing data (1,130 sequences)					
PC6/	SVM	0.8566	0.8592	0.8531	*0.8602*	0.8561	0.7133
PC6/	Random forest	0.8513	0.8869	*0.8053*	0.8973	0.8442	0.7057
**PC6/**	**Deep learning**	**0.8850**	**0.9035**	**0.8620**	**0.9080**	**0.8822**	**0.7707**
Word2Vec/	SVM	*0.7327*	*0.7514*	*0.6956*	*0.7699*	*0.7224*	*0.4668*
Word2Vec/	Random forest	*0.7858*	*0.8549*	*0.6885*	0.8832	*0.7627*	*0.5828*
Word2Vec/	Deep learning	*0.846*	*0.8574*	0.8301	*0.8619*	*0.8435*	*0.6924*

a Top three ranked methods for each index are presented using text formats: first in boldface, second with underline, third in normal text format, and all the rest in italic. ML, machine learning.

Recently, deep learning techniques have been employed in various protein prediction tasks other than AMPs, such as predicting protein-protein interactions (12) and human leukocyte antigen complexes (16), with remarkable performance. As anticipated, both the PC6 and Word2Vec models trained using deep learning outperformed those trained using SVM and random forest. When adopting the encoding method, the performance of PC6 was superior to that of Word2Vec regardless of the machine learning algorithm used or the data set (testing or external) applied. Moreover, model variants using PC6 sequence encoding performed more favorably with the external testing data than with the testing data set. Notably, the performance of the PC6 deep learning method with the external testing data is almost as good as with as the testing data set. All approaches encoded with Word2Vec were less effective than those encoded with PC6 in the external testing set.

### Comparisons among AMP predictors.

**FIG 2:**
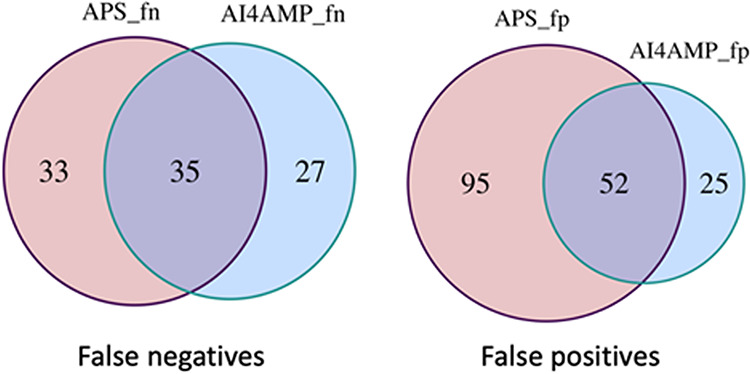
The Venn diagram presents the overlapping of misplaced AMP prediction, false negative (left) and false positive (right), on an external test by PC6/deep learning and Antimicrobial Peptide Scanner vr.2.

We compared our deep learning models encoded by the PC6 method with two other state-of-the-art AMP predictors, namely, APS vr.2 (10) and iAMPpred (9). We prepared external testing by cleaning up sequences listed in each predictor’s training data set and then executing the prediction task on their websites. Therefore, the performance test was unbiased. As shown in Table 3, the PC6 deep learning model was superior to the others for all six measures. A further comparison of the misclassified cases of PC6 deep learning with those of APS vr.2 is presented in Fig. 2. AI4AMP significantly outperformed the other two predictors due to fewer false-positive predictions. To verify that our PC6 encoding method and model architecture provide superior performance and not just require more training data, we trained our model by using the data provided on the APS vr.2 website. We list those external testing results in Table S1 in the supplemental material.

**TABLE 3:** Performance of AMP predictors in an external test set

Predictors	Accuracy	Precision	Sensitivity	Specificity	F1 score	MCC
PC6/deep learning (our platform)	0.8850	0.9035	0.8620	0.9080	0.8822	0.7707
Antimicrobial Peptide Scanner vr.2^*a*^ (embedding layer/deep learning)	0.8097	0.8796	0.7717	0.8601	0.8222	0.6256
iAMPpred^*b*^ (SVM)	0.7367	0.7436	0.7365	0.7368	0.7400	0.4733

a See https://www.dveltri.com/ascan/v2/.

b See http://cabgrid.res.in:8080/amppred/.

10.1128/mSystems.00299-21.2 TABLE S1 The external testing of our PC6-deep learning model trained by fewer data (2,021 AMP and non-AMP) was used in the latest version APS vr.2. Download Table S1, DOCX file, 0.01 MB. Copyright © 2021 Lin et al. 2021 Lin et al. https://creativecommons.org/licenses/by/4.0/ This content is distributed under the terms of the Creative Commons Attribution 4.0 International license.

### AI4AMP can be used in novel AMP designs.

Recently, some research has aimed to generate new AMPs by using computational algorithms or deep learning models. Through these approaches, researchers can create many novel antibiotic peptide candidates. For example, Porto et al. developed an algorithm, Joker, to create new AMP-like variants such as PaDBS1R1 (19), based on non-AMP activity peptides (18). They tested the antibiotic activity of these peptides by using the MIC assay. Nagarajan et al. designed AMPs using deep learning (17) based on a long short-term memory (LSTM) architecture (18) to generate peptide strings and then selected AMP candidates by setting thresholds on peptide charge and amphiphilicity. They employed another Bi-LSTM regression model to score peptides and then derived the optimal 10 sequences and the poorest three MIC assay sequences.

**FIG 3:**
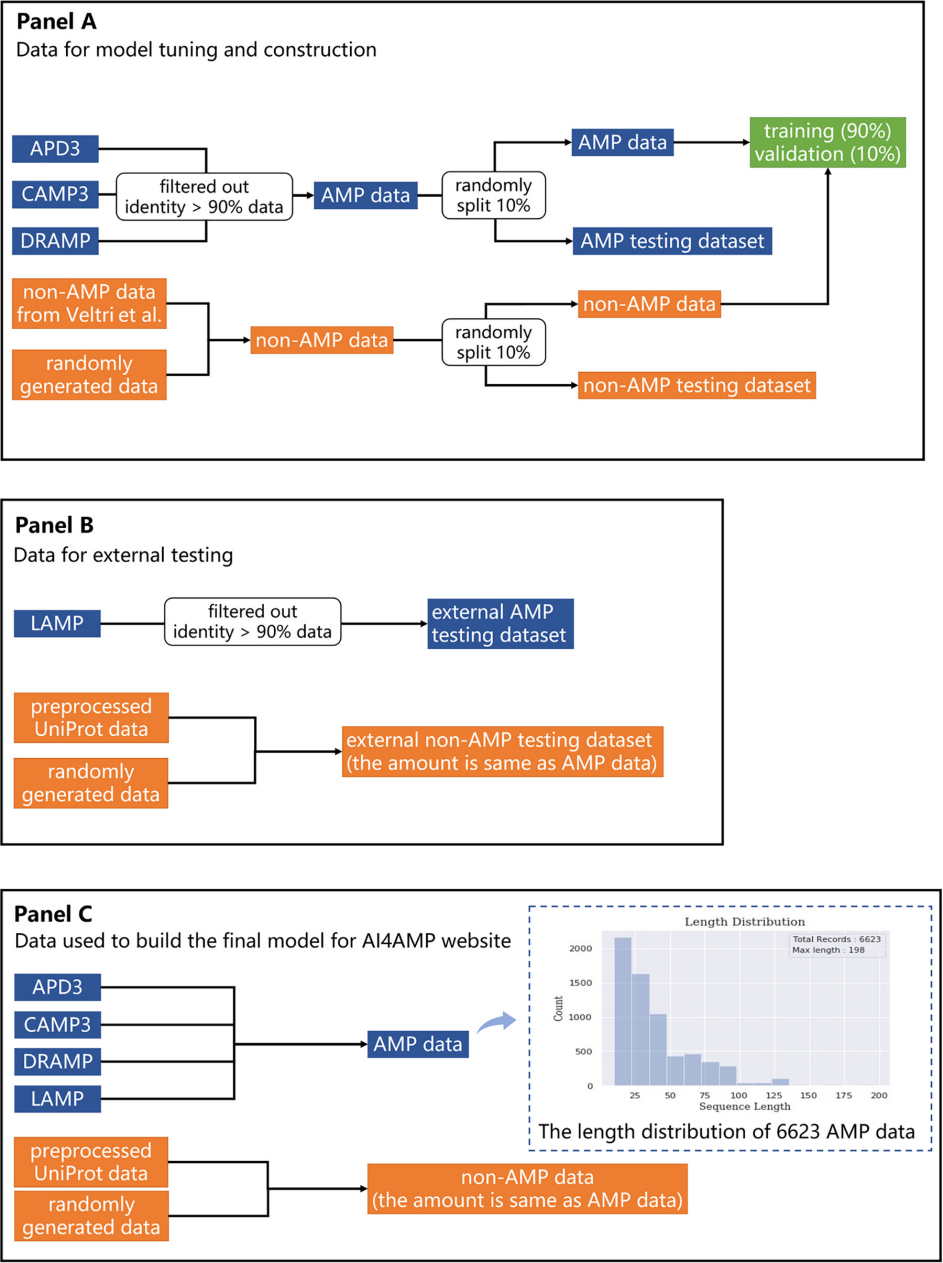
Data processing in this study. (A) Data for model tuning and construction. (B) Data for external testing. (C) Data for the final model.

**FIG 4:**
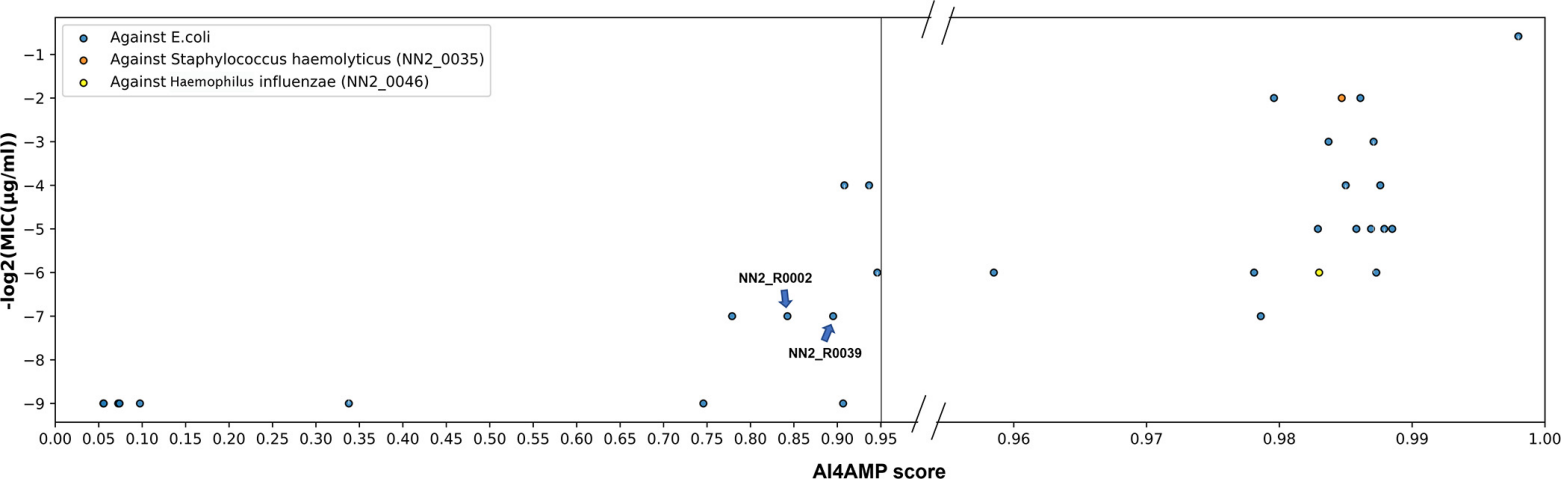
Algorithm-designed peptide AMP activities [−log(MIC)] and their corresponding AI4AMP scores. Plot areas in high AI4AMP scores (>0.95) were expanded to show the crowd data points. MICs beyond the testing range (>128 or >256 μg/ml) were assigned to 512 [e.g., −log_2_(512) = −9].

To implement the web application AI4AMP, we trained our PC6 deep learning model with 13,246 peptide sequences (6,623 AMP + 6,623 non-AMP sequences [Fig. 3C]) with all parameters optimized previously. Then, we calculated the AI4AMP score in relation to synthetic peptides from Joker (19) and the LSTM model (17) and summarized the scores from AI4AMP and MIC (Fig. 4). Peptides and numeric values represented in Fig. 4 are listed in Tables S2 and S3.

10.1128/mSystems.00299-21.3 TABLE S2 The lower MIC against E. coli and S. aureus of synthetic peptides in Joker (19) received higher AI4AMP scores in general. Download Table S2, DOCX file, 0.02 MB. Copyright © 2021 Lin et al. 2021 Lin et al. https://creativecommons.org/licenses/by/4.0/ This content is distributed under the terms of the Creative Commons Attribution 4.0 International license.

10.1128/mSystems.00299-21.4 TABLE S3 The 10 top-scored synthetic peptides predicted by LSTM model in the work of Nagarajan et al. (17) received high AI4AMP scores. In the original publication, two peptides did not find AMP activities against E. coli (MIC > 128) but proved to be AMP against other pathogens. Besides, two out of the three worst-Bi-LSTM-scored peptides still showed moderate AMP activities (NN2_R0002 and NN2_R0039, MIC = 128 μg/ml); they gained scores of ∼0.85 by AI4AMP. The Bi-LSTM scores used in the study fluctuated and were less helpful in predicting AMP potential. Download Table S3, DOCX file, 0.02 MB. Copyright © 2021 Lin et al. 2021 Lin et al. https://creativecommons.org/licenses/by/4.0/ This content is distributed under the terms of the Creative Commons Attribution 4.0 International license.

The synthetic peptides obtained from Joker with a lower MIC against Escherichia coli and Staphylococcus aureus received higher AI4AMP scores (Table S2). Furthermore, the 10 top-scoring peptides reported by Nagarajan et al. (17) received high AI4AMP scores, among which two peptides did not show AMP activity against E. coli (MIC > 128 [Table S3]). Although these two peptides did not demonstrate the potential to kill E. coli, they reportedly proved their AMP activities against other pathogens such as Staphylococcus haemolyticus (NN2_0035 killing activity: MIC = 4 μg/ml) and Haemophilus influenzae (NN2_0046 killing activity: MIC = 64 μg/ml). Moreover, two of the three poorest-performing peptides on Bi-LSTM scores showed moderate AMP activities (NN2_R0002 and NN2_R0039: MIC = 128 μg/ml, AI4AMP = ∼0.85). The Bi-LSTM scores used in the study fluctuated and were unhelpful in the prediction of AMP potential.

Presently, determining a precise AI4AMP score cutoff is difficult. Although we used the receiver operating characteristic (ROC) curve and determined the optimal AI4AMP threshold to be approximately 0.41 (the ROC curve plot is displayed in Fig. S1), Joker-designed variants tended to have higher AI4AMP scores than did those obtained from the original template as well as demonstrating a correlation with bacterial killing performance. Various AMP modes of activity have been proposed, and all these known peptides are included in the up-to-date AMP data set for AI4AMP model training. Thus, a peptide with a high score represents a possible candidate for some other microbe taxa. In summary, peptides with higher AI4AMP scores are more likely to exhibit significant antimicrobial activity.

10.1128/mSystems.00299-21.1 FIG S1 The ROC curve plot of AI4AMP. We calculated the geometric mean for each threshold and then found the best threshold at 0.4091(geometric mean is 0.923). Download FIG S1, TIF file, 0.09 MB. Copyright © 2021 Lin et al. 2021 Lin et al. https://creativecommons.org/licenses/by/4.0/ This content is distributed under the terms of the Creative Commons Attribution 4.0 International license.

### AI4AMP as web portal for novel AMP identification.

**FIG 5:**
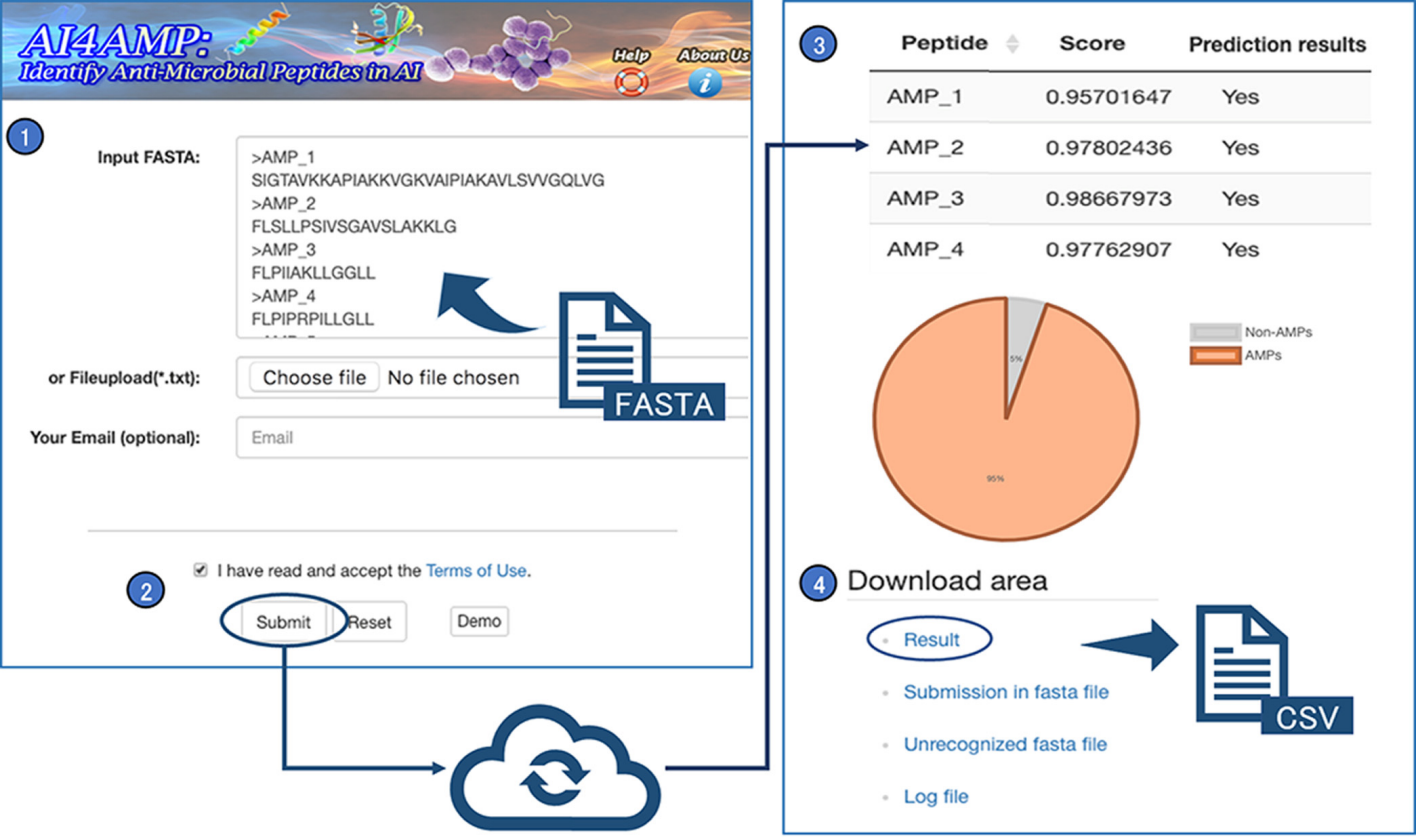
AI4AMP web server. The left panel shows the user interface (UI) input. (1 and 2) Users may either copy-paste sequences in FASTA format directly in the “input FASTA” form or upload a FASTA file to “Fileupload (*.txt).” Users may submit the query with a valid email address in “Your Email” to trace back to the job result page or stay on the UI for redirecting to the output. (3 and 4) Users may browse the scores and prediction results on the result page (the right panel) and retrieve the output CSV file in the download area. A pie chart summarizes the proportion of predicted AMPs and non-AMPs in the input sequences.

AI4AMP, developed in this study, is a website service that provides a user interface to execute the PC6 deep learning model to predict whether peptide sequences are potential AMPs. Its input is protein sequence data in the FASTA format and IUPAC single-letter mode. Notably, our prediction model accepted only input peptides in 20 standard amino acid residues. The prediction did not proceed if input sequences contained unusual amino acids such as B, Z, U, O, J, I, n, “-,” and X. For an input sequence longer than 200 residues, a sliding window with a width of 200 and a step size of 100 was applied to the sequence, converting the original one into multiple sequences; then, the jobs proceeded independently for each substring. The AI4AMP user interface is presented in Fig. 5. AI4AMP output is a two-column CSV file describing scores (ranging from 0 to 1) and prediction results (*yes* or *no*) of each input sequence (including each substring of a long sequence). The score represented a measure of AMP tendency. The AMP-based judgment (the prediction result) was based on the prediction score with a threshold of 0.5.

## DISCUSSION

First, we proposed a new protein-encoding method, PC6. This method captured some of the physicochemical properties of peptides. We demonstrated that different protein-encoding methods markedly affected the performance of a prediction model. This PC6 method could be adopted for tasks that employ the peptide side chain’s basic biochemical features (H1, V, Pl, pI, pK_a_, and NCI) for encoding. Furthermore, assembling a small training data set is always challenging in machine learning approaches. The property-encoding method PC6 and a demarcated external data set and training data set helped us develop a robust model for capturing essentials for prediction and avoiding the trap of overfitting. We demonstrated that the PC6 deep learning approach was superior to other approaches. Finally, we implemented this model on a user-friendly website, AI4AMP. This implementation can serve as a beneficial tool for drug developers who can use the tool to discern AMPs and non-AMPs. In AI4AMP, we focused on the prediction of antibacterial peptides.

Updated versions are expected in the future as the training data are updated to reflect newly discovered AMPs, thus further improving our prediction model. In addition, the training model for specific strains, such as various multidrug-resistant, Gram-positive or Gram-negative bacteria, will be developed according to the increasing number of well-characterized AMPs to provide a more accurate prediction based on strategies similar to those used in this study.

## MATERIALS AND METHODS

### Data collection.

The AMP data set was obtained from four databases: APD3 (20), LAMP (21), CAMP3 (22), and DRAMP (23). We downloaded all antibacterial AMP data from the four databases, excluding AMPs with sequence lengths shorter than 10 amino acids and those containing unusual amino acids, such as B, Z, U, X, J, O, i, n, and “-.” After removing duplicate records, we finally obtained 6,623 sequences for the AMP data set. Figure 3C shows the data set’s length distribution; notably, most AMPs are <50 amino acids in length.

The non-AMP data set was a combination of real-world peptides and artificially generated sequences. Real-world peptides were obtained from the UniProt database (24) by using the following inclusion criteria: (i) sequence length between 10 and 50 amino acids and (ii) without the use of AMP-related keywords such as “antimicrobial,” “antibiotic,” “amphibian defense peptide,” and “antiviral protein” in its annotation. Artificially generated sequences were randomly derived from 20 essential amino acids, and their length distribution was the same as those in the AMP data set. We eventually obtained a non-AMP data set of 6,623 sequences. Our design thus established balanced AMP and non-AMP data input for deep learning model training and testing processes.

### Data for model tuning and construction.

We used 3,528 AMP sequences (AMP_3528) and 3,528 non-AMP sequences (non_AMP_3528) to construct and refine our model. AMP_3528 was obtained from the original AMP data set (with 6,623 sequences), excluding data from the LAMP database and sequences with high similarity (>90% by CD-HIT [25]) to this set. In non_AMP_3528 (3,528 sequences), 1,778 sequences were obtained from the data set used by Veltri et al. (10); the remaining 1,750 sequences were randomly selected from the non-AMP data set described previously, thereby resulting in equally sized AMP and non-AMP input data sets. Furthermore, the 7,056 sequences (AMP_3528 + non-AMP_3528) were stratified for model building and testing; 706 sequences (10%) were reserved as the test set, and the other 6,350 sequences were divided into a training set (5,715 sequences) and validation set (635 sequences) (Fig. 3A).

### Data for external testing.

An external AMP testing data set (a total of 1,130 sequences) was composed of 565 LAMP database AMPs (excluding sequences sharing ≥90% sequence identity) and 565 non-AMP sequences (285 non-AMP short peptides randomly selected from the UniProt database and 280 randomly generated sequences); the peptide length distribution of these AMPs was the same as that of collected AMPs (Fig. 3B).

### The PC6 encoding method.

**FIG 6:**
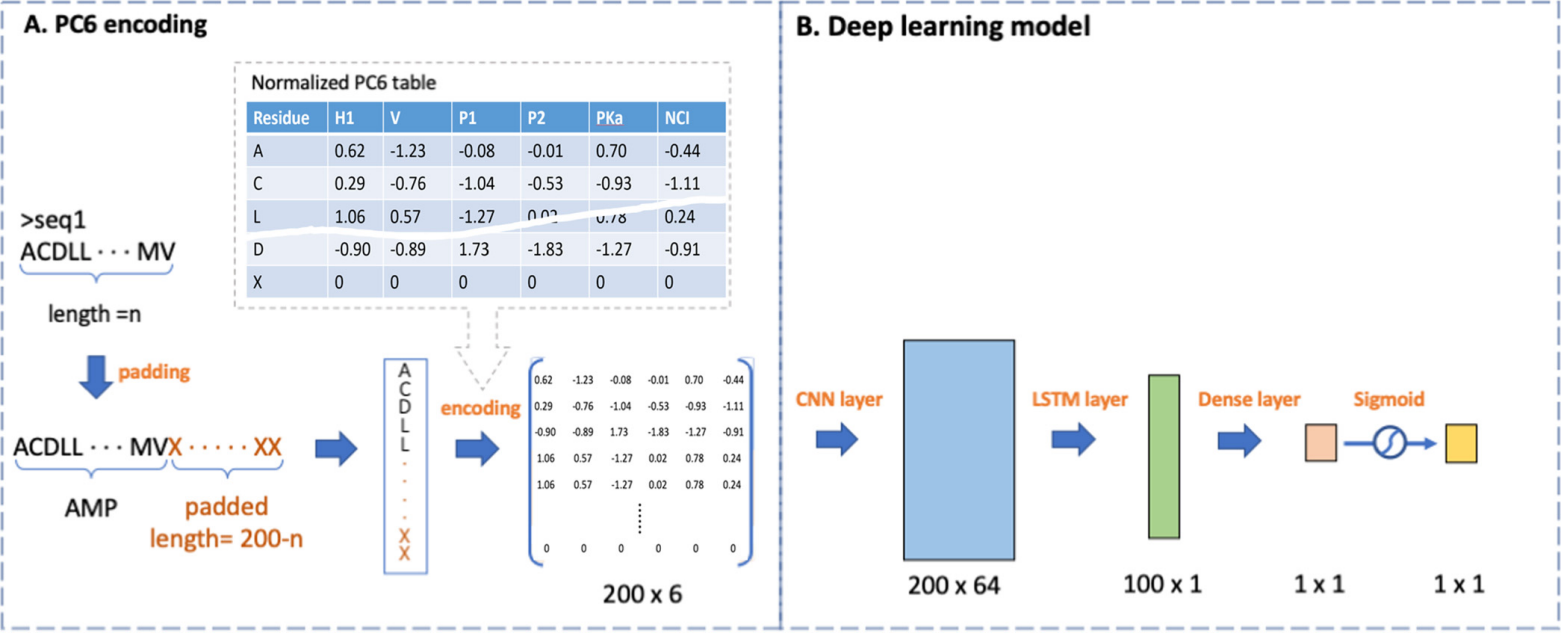
(A) PC6 protein-encoding method. Each input sequence will be transformed into a 200 × 6 matrix, respectively. (B) Deep neural network model. The PC6 encoded data matrix will pass through one convolution layer, one LSTM layer, and one dense layer.

The core idea of the PC6 encoding method is to apply word embedding in relation to the physicochemical properties of each amino acid (Fig. 6A). We first derived a table of 20 amino acids with six corresponding physicochemical properties. We normalized the value of the 20 amino acids for each property to prevent the improper weighting effect caused by differences in the numerical range. One extra character, “X” with 0 for all six properties, was added for sequence padding. Thus, a protein-encoding table containing 21 tokens for the 20 amino acids (plus one padding character) was generated. Because the AMP data set sequences had a maximum length of 198 amino acids, we padded all sequences in the collection to 200 amino acids in length. Subsequently, each sequence formed a 200-element vector and was transposed into a column vector starting as a token string. After that, we replaced each token with the PC6 protein-encoding table and formed a 200 × 6 matrix for each input sequence.

The encoding method of the physicochemical component (PC7) is the same as that of PC6 except for seven properties proposed in the original autocovariance (AC) method for encoding, and a 200 × 7 matrix is derived for each sequence accordingly.

### Deep neural network model.

We implemented our neural network by using the Keras application programming interface. The model architecture of natural language processing (NLP) tasks typically consists of a convolutional layer, a long short-term memory (LSTM) layer, and a dense layer (Fig. 6B). We did not apply the pooling layer in our model because it may remove intermediate features, causing loss of information regarding some protein fragments, as noted in DeeperBind (26). The convolutional layer was developed using 64 one-dimensional filters of 16 units in length; it used a rectified linear unit activation function and an LSTM layer containing 100 units. In addition, only one convolutional layer was used because adding more convolutional layers decreased the validation accuracy in our case. This result accorded with that of the training protein-protein interaction model reported by Sun et al. (12). Binary cross entropy was implemented in the loss function. The Adam optimizer, with a learning rate of 0.0003, was applied. The output layer comprised a one-dimensional dense layer with a sigmoid activation function that produced a value ranging from 0 to 1, indicating the relatedness of AMPs. We trained, tuned, and tested our model and further challenged it with the external testing data set. The stratification of the collected AMPs and non-AMPs is described in Materials and Methods and in Fig. 3A and B. Grid search was employed for hyperparameters including the adjustment of the learning rate, batch size, and optimizer.

We used all available data (including 6,623 AMP and 6,623 non-AMP sequences) to train the final model, which was housed on our AI4AMP website. The final model was trained for 200 epochs, and the batch size was set to half the number of the training data set. To prevent overfitting, we stopped the training early.

### Model evaluation.

To evaluate the performance of model variants, we determined the accuracy, precision, sensitivity, specificity, F1 score, and Matthews correlation coefficient (MCC) as the rating scores of binary classification methods. We used the Python package “scikit-learn” (27) to calculate these metrics from true positive (TP), true negative (TN), false positive (FP), and false negative (FN). The functions of the metrics are defined as follows:

$$\mathrm{Accuracy}=\frac{\mathrm{TP}+\mathrm{TN}}{\mathrm{TP}+\mathrm{TN}+\mathrm{FP}+\mathrm{FN}}$$

$$\text{Precision = }\frac{\text{TP}}{\mathrm{TP}+\mathrm{FP}}$$

$$\text{Sensitivity = }\frac{\text{TP}}{\mathrm{TP}+\mathrm{FN}}$$

$$\text{Specificity = }\frac{\text{TN}}{\mathrm{TN}+\mathrm{FP}}$$

$$\text{F1 score = }\frac{\text{2TP}}{\mathrm{2TP}+\mathrm{FP}+\mathrm{FN}}$$

$$\text{MCC = }\frac{\text{TP }\times \text{ TN }-\text{ FP }\times \text{ FN}}{\sqrt{\left(\mathrm{TP}+\mathrm{FP}\right)\left(\mathrm{TP}+\mathrm{FN}\right)\left(\mathrm{TN}+\mathrm{FP}\right)\left(\mathrm{TN}+\mathrm{FN}\right)}}$$

### AI4AMP implementation.

After confirming the optimal model architecture, hyperparameters, and protein-encoding methods, we trained the model with all available data (including 6,623 AMP and 6,623 non-AMP sequences [Fig. 3C]).

### Data availability.

AI4AMP is freely accessible at http://symbiosis.iis.sinica.edu.tw/PC_6/. All the data sets used in this study are available in the online HELP at https://symbiosis.iis.sinica.edu.tw/PC_6/helppage.html. The source code of the PC6 encoding method and the trained deep learning used in the AI4AMP model are available at https://github.com/LinTzuTang/PC6-protein-encoding-method and https://github.com/LinTzuTang/AI4AMP_predictor.
