# Supplementary material for: AI4AMP: an Antimicrobial Peptide Predictor Using Physicochemical Property-Based Encoding Method and Deep Learning
Source: mSystems. 2021 Nov 16;6(6):e00299-21. doi: 10.1128/mSystems.00299-21 (PMC8594441; doi:10.1128/mSystems.00299-21)
Supplement: TABLE S1 [file msystems.00299-21-st001.docx]

| predictors | Accuracy | Precision | Sensitivity | Specificity | F1 score | MCC |
| --- | --- | --- | --- | --- | --- | --- |
| PC6/ AI4AMP | **0.8619** | 0.8460 | **0.8850** | 0.8389 | **0.8651** | **0.7247** |
| APS vr.2 | 0.8097 | 0.8796 | 0.7717 | 0.8601 | 0.8222 | 0.6256 |
