# Supplementary material for: AI4AMP: an Antimicrobial Peptide Predictor Using Physicochemical Property-Based Encoding Method and Deep Learning
Source: mSystems. 2021 Nov 16;6(6):e00299-21. doi: 10.1128/mSystems.00299-21 (PMC8594441; doi:10.1128/mSystems.00299-21)
Supplement: TABLE S2 [file msystems.00299-21-st002.docx]

| Peptide | Sequence | MIC against *E. coli* (μg/ml) | MIC against *S. aureus* (μg/ml) | AI4AMP score |
| --- | --- | --- | --- | --- |
| PaDBS1R1 | PKILNKILGKILRLAAAFK | 1.5 | 3 | 0.998 |
| EcDBS1 | MKKLFAALALAAVVAPVW | >256 | >256 | 0.7458 |
| EcDBS1R4 | PMKKKLAARILAKIVAPVW | 16 | >256 | 0.985 |
| EcDBS1R8 | PMKKLFAAKILARVVAKIW | 32 | >256 | 0.9879 |
| MmDBS1 | KKINNTILSGLDSVASFS | >256 | >256 | 0.0975 |
| MmDBS1R5 | PKKINKLLLRILDKIASFS | 32 | n/t | 0.9829 |
| MmDBS1R9 | PKKINNTILKLLDRVASKI | 64 | n/t | 0.9585 |
| PaDBS1 | MARNKPLGKKLRLAAAFK | >256 | >256 | 0.9066 |
| PaDBS1R2 | PMKLLKRLGKKIRLAAAFK | 32 | n/t | 0.9869 |
| PaDBS1R5 | PMARNKILGKILRKIAAFK | 8 | 64 | 0.9871 |
| PaDBS1R6 | PMARNKKLLKKLRLKIAFK | 16 | 128 | 0.9365 |
| PcDBS1 | MNAINFTCTVHKKVAISV | >256 | >256 | 0.0724 |
| PcDBS1R1 | PKLAIRITCKIHKKVAISV | 64 | 256 | 0.946 |
| PcDBS1R5 | PMNAIKLLCRVHKKIAISV | 128 | n/t | 0.9786 |
| PyDBS1 | NRESQFYQSRRKKLSSQV | >256 | >256 | 0.0553 |
| PyDBS1R8 | PNRESQFYKILRKILSKIV | 16 | 64 | 0.9081 |
| PyDBS1R9 | PNRESQFYQKLLKKLSSKI | >256 | 256 | 0.3378 |
| TcDBS1 | MSAEVVVKKVDGDALELG | >256 | >256 | 0.0739 |
| TcDBS1R2 | PMKLLVRVKKKIGDALELG | 128 | n/t | 0.7789 |
