# Supplementary material for: AI4AMP: an Antimicrobial Peptide Predictor Using Physicochemical Property-Based Encoding Method and Deep Learning
Source: mSystems. 2021 Nov 16;6(6):e00299-21. doi: 10.1128/mSystems.00299-21 (PMC8594441; doi:10.1128/mSystems.00299-21)
Supplement: TABLE S3 [file msystems.00299-21-st003.docx]

| Peptide | Sequence | MIC against *E. coli* (μg/ml) | AI4AMP score |
| --- | --- | --- | --- |
| Top-scored peptides in Bi-LSTM-scored approach | | | |
| NN2_0018 | YLARAIRRTLARLLL | 32 | 0.9858 |
| NN2_0022 | EWRVARRAVQRLRHLARRYH | 16 | 0.9876 |
| NN2_0024 | ALKKMLRLAKRLS | 64 | 0.9781 |
| NN2_0027 | VLSAFHKVIKIIHHISHF | 32 | 0.9885 |
| NN2_0029 | RKFRKILHRARKWI | 8 | 0.9837 |
| NN2_0035 | RRWGRWHRMRRRGR | >128* | 0.9847 |
| NN2_0039 | FWKGLVKAAFKIVHAGS | 64 | 0.9873 |
| NN2_0046 | GWKAIHKAAKGIHTYVN | >128** | 0.983 |
| NN2_0050 | SWKKFFKKARSLPKLF | 4 | 0.9861 |
| NN2_0055 | YKRWKKWRSKAKKIL | 4 | 0.9796 |
| Worst-scored peptides in Bi-LSTM-scored approach | | | |
| NN2_R0002 | KWKCLAKVGIAAH | 128 | 0.8426 |
| NN2_R0039 | KRSWDIVKKYVGVVVGTIH | 128 | 0.8951 |
| NN2_R0048 | AGEKRIIKKIDEAFQ | >128 | 0.0558 |

*: NN2_0035 with outstanding killing activity (MIC = 4 μg/ml) on *Staphylococcus haemolyticus*

**: NN2_0046 with moderate activity (MIC = 64 μg/ml) on *Hemophilic influenzae*
