# Supplementary figures and images for: AI4AMP: an Antimicrobial Peptide Predictor Using Physicochemical Property-Based Encoding Method and Deep Learning
Source: mSystems. 2021 Nov 16;6(6):e00299-21. doi: 10.1128/mSystems.00299-21 (PMC8594441; doi:10.1128/mSystems.00299-21)

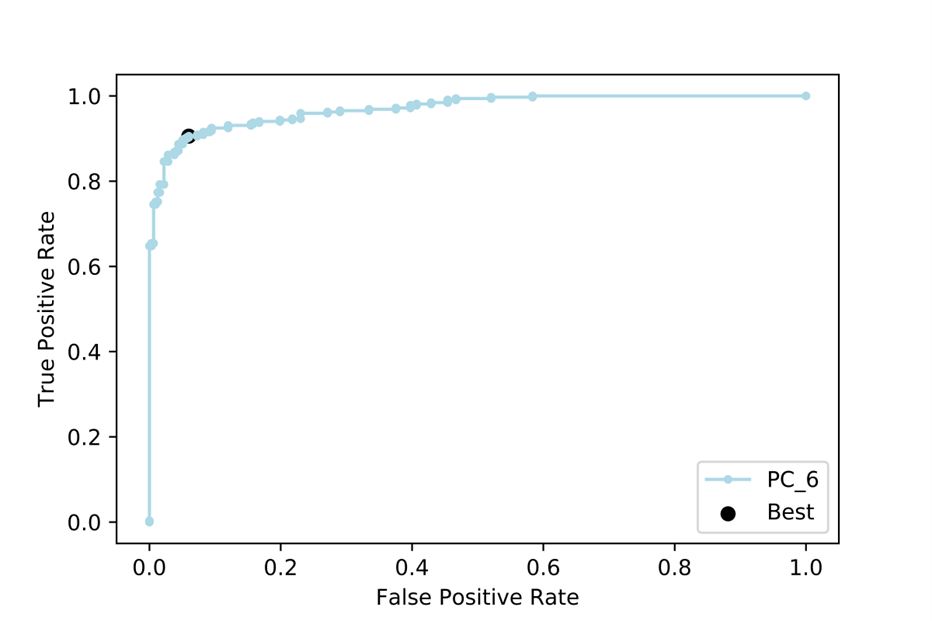

Supplement: FIG S1 [file msystems.00299-21-sf001.tif]
